# Integration of SMP with PVDF Unimorph for Bending Enhancement

**DOI:** 10.3390/polym13030415

**Published:** 2021-01-28

**Authors:** Sudarshan Kalel, Wei-Chih Wang

**Affiliations:** 1 Institute of Nanoengineering and Microsystems, National Tsinghua University, Hsinchu City 300, Taiwan; sdrshnkalel9@gmail.com; 2 Department of Power Mechanical Engineering, National Tsinghua University, Hsinchu City 300, Taiwan; 3 Department of Mechanical Engineering, University of Washington, Seattle, WA 98195, USA; 4 Department of Electrical & Computer Engineering, University of Washington, Seattle, WA 98195, USA

**Keywords:** PVDF, unimorph, SMP, self-heat generation

## Abstract

Heat generation in active/passive layer-based piezoelectric actuators is unavoidable due to the mechanical, dielectric, and resistive losses in the material. In this work, a polyvinylidene fluoride (PVDF)-based unimorph cantilever actuator is developed with simulation and experimental studies on the effect of DC high voltages on heat production in the PVDF layer. A layer of one-way shape memory polymers (1W-SMPs) is integrated in the actuator to exploit the heat produced to increase the bending angle. The length and mounting location of the SMP layer impacts the bending of the actuator; by using an SMP layer with a length equal to half of the PVDF layer at the center of the unimorph actuator, the absolute bending angle is increased to 40° compared to the base piezo bending angle of 4° at 20 V/µm.

## 1. Introduction

Polyvinylidene fluoride (PVDF) is a dielectric material that exhibits piezoelectric, pyroelectric [1], and photoelectric [2] effects, making it a popular choice among electroactive polymers [3,4,5]. The lower piezo coefficient (d31) of PVDF compared to widely used lead zirconate titanate (PZT) material offers low actuation due to which copolymers and terpolymers of PVDF have been used to enhance this effect [5,6,7,8]. The high operating voltage, low electrical conductivity of PVDF, and mechanical and dielectric losses are responsible for heat generation in piezoelectric materials [9,10,11,12,13]. The imaginary part of the dielectric constant of a material, a function of frequency, accounts for conductance and promotes self-heat generation [10,11]. In the case of active and passive layer-based actuators, this generated heat is transferred to the surroundings by radiation and convection from the active surface, while some of the heat is transferred to the adjacent passive layers through conduction. The adhesive layers used for bonding are not perfect insulators either. So, the heat can significantly contribute to the actuation process and cannot be neglected.

The current work investigates the amount of heat generated within the piezoelectric layer and its contribution to the bending of the unimorph actuator. A method is also proposed for utilization of this heat to improve actuator performance which usually affects it adversely [10]. The use of temperature-sensitive deforming materials to enhance the bending of the unimorph actuator would achieve an effective way to utilize this heat. The shape memory polymers are stimuli-responsive materials where heat can be used as an external stimulus to switch between the temporary and permanent shapes of the material [14,15]. Polyurethane-based shape-memory materials with their unique crystal structures provide high recovery strains at low transition temperatures (T_g_) [16,17]. Poly (caprolactone) (PCL)- and poly (ester urethane)-based SMPs with transition temperatures near to atmospheric and human body temperatures have been widely used for many applications in recent works [18,19]. In the present work, a one-way shape-memory polyurethane material is used to exploit the heat and enhance the bending of unimorph. The methodology section ahead describes the preparation method for SMP films and the fabrication of a layer-based cantilever actuator. The verification of heat generated in the piezo layer and its effect on the bending of the actuator acting as thermal bimorph is presented in the results section. A method to achieve maximum bending of the integrated SMP/PVDF actuator for the applied DC field is also discussed.

## 2. Materials and Methods

For fabrication of cantilever unimorph, 50 µm metalized (80 nm Cu and 20 nm Ni) PVDF film was purchased from PolyK Technologies, Philipsburg, PA, USA, to use as the active layer. The bending in the case of unimorph configuration is achieved due to the difference in the mechanical resistance offered by two layers to the deformation; hence, the passive layer plays a vital role. Polyimide (Kapton tape), Scotch tape [7], and Teflon were compared through the simulation study. Scotch tape was found to be the effective choice, with its density, elastic modulus, and Poisson ratio close to that of PVDF. So, the transparent type 60 µm thick Scotch tape by 3M Technologies was used as a passive layer.

The self-heat generation within the piezo layer by resistive heating was analyzed in COMSOL Multiphysics to obtain the temperature corresponding to the applied electric field (DC). Properties of materials used in the simulations are listed in Table 1. Convective type heat flux with a coefficient of 5 W/m^2^K (natural heat convection) and surface-to-ambient radiation coefficient of 0.8 (1 being the ideal case) with an ambient temperature of 300 K were used in the study. The surface temperature of the unimorph actuator was measured experimentally by using FLIR camera imaging, with surrounding temperature of 27 °C. Since the PVDF is pyroelectric and photoelectric, high-temperature and high-intensity light sources were avoided in the surroundings.

For SMP/PVDF integration study, MS-4520 solution type shape-memory polyurethane with 45 °C T_g_ was purchased from SMP Technologies, Tokyo, Japan. The SMP solution was diluted with DMF to produce 100 µm thin films by a screen-casting technique and cast films were cured at 120 °C for 4 h. The developed SMP film was programmed with 30% strain and was mounted on the PVDF/Scotch tape unimorph using adhesive transfer tape. 3M 467MP adhesive transfer tape was used for layer bonding. This flexible adhesive does not offer mechanical resistance and there is a lack of heat transfer in the structure. The SMP programming was carried out by first heating the SMP to T_g_, applying a tensile load, followed by cooling down the SMP to room temperature under the load. The prebending of unimorph was observed after mounting the SMP layer at no voltage due to the stress induced during the programming. The bending of the cantilever actuator was captured by a video camera system (DFK33UX252 by Imaging Source) and performance was characterized by the bending angle of the actuator, as explained in the discussion section.

## 3. Results and Discussion

### 3.1. Temperature vs. Electric Field

**Figure 1:**
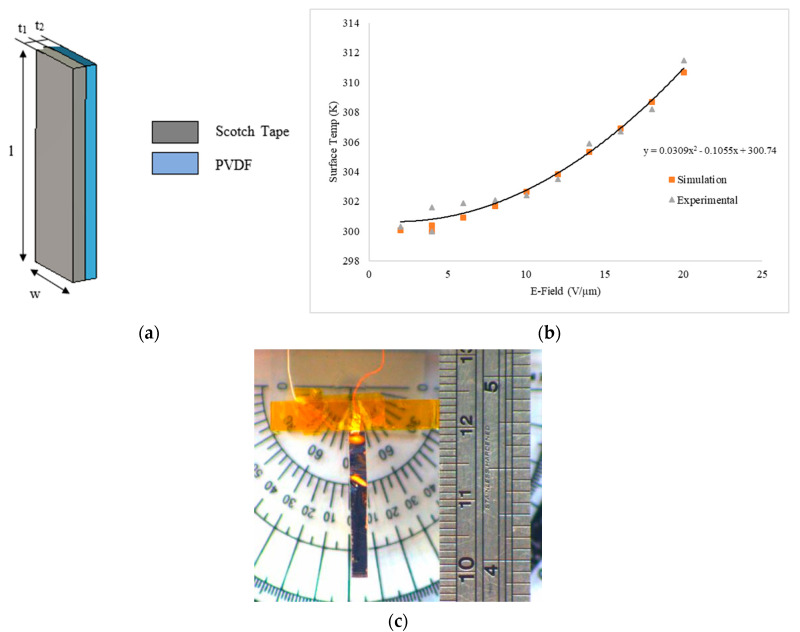
(**a**) Schematic representation of cantilever unimorph actuator used for self-heat generation study, l = 20 mm, w = 2.5 mm, t_1_ = 60 µm, t_2_ = 50 µm. (**b**) A plot of average surface temperature as a function of applied E-field. (**c**) The polyvinylidene fluoride (PVDF)/Scotch tape unimorph experiment setup. Polyimide (PI) tape was applied at the wire and PVDF connections to provide additional heat and electrical insulation.

The effect of the applied electric field on the surface temperature of unimorph is shown in Figure 1. The amount of heat generated in the PVDF layer is dependent on the electrical resistance offered by the polymer crystalline structures. This intrinsic resistance (inversely proportional to its electric conductivity) combined with the naturally occurring small leakage current is suspected to be the cause in of this small joule heat generated in the PVDF, which ended up increasing proportionally with the applied electric field. The minor deviations in the temperature values obtained by simulation and experiment results might be due to the fact that the conduction effect between the layers during the simulation was ruled out, as were the actual heat convection coefficient and imperfect surrounding conditions that increase the temperature of the layer due to photo and pyroelectric effects, and calibration errors in IR imaging system. The increase in the surface temperature of the actuator at lower electric fields is comparatively less than the higher field regions. After the curve fitting, the increase in temperature is proportional to the square of the applied electric field as power loss is always proportional to the square of applied voltage [9]. According to the simulation study, when heat is distributed over the surface of the layer, the temperature is maximum at the center, whereas, in the experimental study, most of the heat is concentrated at the fixed end of the cantilever near the electrical contacts due to nonideal electrode techniques. To avoid errors in the experimental results, the temperature at the different locations on the actuator surface was measured and an average value was obtained.

Most of the previous studies ignore the effect on the actuation of the cantilever unimorph due to the heat generated in the piezo layer. Due to the difference in coefficients of thermal expansion, these layers exhibit a thermal bimorph effect. Through the simulation studies as depicted in Table 2, it was found that this heat effect significantly contributes to the deformation of actuators; therefore, in reality, all the achieved deformations are a combined result of the piezoelectric and thermal induced stress effects.

### 3.2. Integration of SMP with PVDF Unimorph

**Figure 2:**
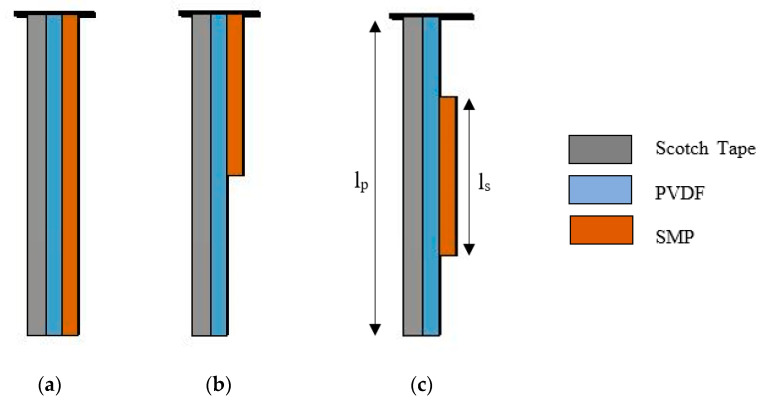
Different configurations utilized for maximum tip deflection of cantilever actuator (Table 3). l_p_ = length of PVDF layer = 20 mm, l_s_ = length of SMP layer, (**a**) Sample 1 → l_s_ = l_p_, (**b**) Sample 2 → l_s_ = l_p_/2 at l_p_ = 0 (at fixed end), and (**c**) Sample 3 → l_s_ = l_p_/2 at l_p_/2 (at center).

In this section, use of the SMP layer for the exploitation of heat generated in the PVDF/Scotch tape unimorph is discussed. Though the transition temperature of SMP is 45 °C, the memory effect could be observed at a steadily increasing rate, starting 7° below the T_g_. From the temperature measurement, it is safe to assume that the heat generated within PVDF is enough to actuate the programmed SMP layer mounted on the unimorph. When the applied field reached 20 V/µm, and after 60 s, a temperature of around 37 °C was achieved, which is capable of inducing the memory effect. This effect was utilized to increase the deformation of the unimorph actuator. As noted in Table 3, the bending of the actuator due to the piezoelectric effect alone was not more than 4° at 20 V/µm. However, the additional actuation from the SMP was found to be due to the heat from the PVDF, and it is highly dependent on the dimension and placement of the SMP relative to the overall structure (Figure 2). Samples 1, 2, and 3 depict the location and length of the SMP used on an earlier developed unimorph. The length and location of the SMP layer affect the weight of the structure and blocking force developed in the actuator responsible for the bending.

In Sample 1, as shown in Figure 2, when the length of the SMP layer is equal to the length of the PVDF, the self-weight of the structure increases. This results in a bending angle of 4° at 20 V/µm due to piezoelectric effect and 12° with combined SMP enhancement (shown in Table 3). Rather than using SMP over the full layer, the SMP can only be mounted near the fixed end to reduce the weight of the actuator. In Sample 2, the SMP was fabricated with a length half of the PVDF layer and mounted at a fixed end (Figure 2). The enhancement in bending of the unimorph was low (4° with piezo effect and 13° with combined SMP and piezo effect) due to the shorter SMP active length (Table 3). To achieve the maximum bending, Sample 3 was fabricated where the SMP layer was equal to half of the length of PVDF and mounted at the center of the PVDF layer. The force developed at the center of the layer induces a larger bending (Δθ = 40°) due to SMP, while the piezo effect remains the same (Δθ = 4°) (Table 3).

**Figure 3:**
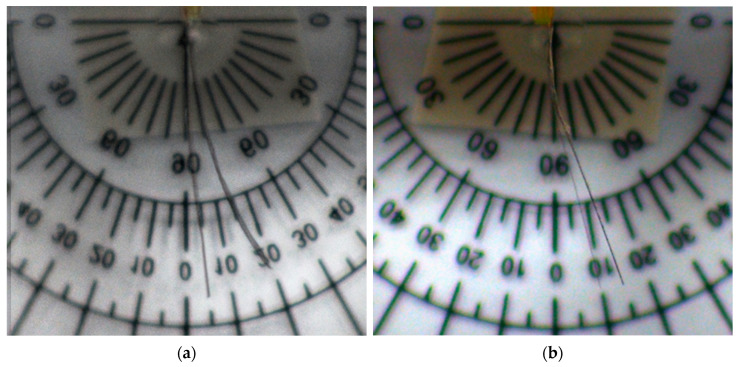

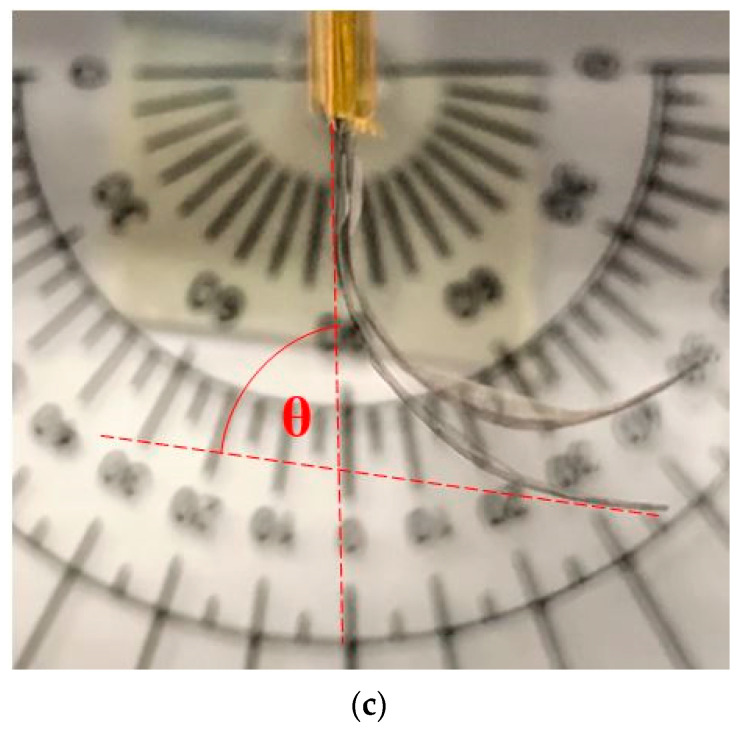
Bending of actuator showing bending enhancement before and after SMP/piezo actuation. (**a**) Sample 1 → l_s_ = l_p_, (**b**) Sample 2 → l_s_ = l_p_/2 at l_p_ = 0, and (**c**) Sample 3 → l_s_ = l_p_/2 at l_p_/2. Bending angle (θ): the exterior angle between two ends of the actuators.

The location of the SMP layer also affects the initial stage of the actuator. For Samples 1 and 2, the prebending angles are small (3° and 8°, respectively). However, Sample 3 has a higher prebending angle of 80°. Figure 3 shows the results of all three samples, where the initial and final bending angles (with both piezo and SMP effects are included) are shown. The corresponding bending angles are summarized in Table 3. It is obvious from the results of the experiment that the first two designs do not display any larger tip deflections or bending angles. On the other hand, the design of Sample 3 shows a large improvement of the bending angle, by 40° (Table 3), and a relatively large (0.83 mm) tip displacement (Table 4) after SMP/piezo actuation.

The tip deflection and bending curvature of different cantilever PVDF actuators, along with reference, are summarized in Table 4. The proposed Scotch tape–PVDF–SMP layer-based actuator shows a much better bending performance than other reported PVDF actuator designs [4,6,7,8]. With the proposed Sample 3 design, the actuator produces a larger tip deflection (0.83 mm) and a smaller 104 m^−1^ bending curvature. In terms of tip deflection, a 20 µm thick PVDF unimorph with smaller geometry and moment of inertia that operates at 15 V/µm (reported in reference [4]) produces only a 0.1 mm tip displacement. Though a 0.3 mm deflection is obtained with relatively smaller E-field of 3.75 V/µm (shown in reference [8]), the actuation is performed with a much larger geometry. For bending curvature, the actuators developed by [6] and [7] with about the same order of moment of inertia demand much higher operating electric fields (>70 V/µm) and larger geometries to produce much larger bending curvatures (140 and 150 m^−1^, respectively).

Since the enhancement of the bending angle only occurs in a single direction, after the removal of the electrical field, it is hard to return the actuator to its initial position. To solve this problem, a two-way SMP can be implemented, where with an increase in the temperature (by increase in DC bias) up to the T_g_, the actuator is bent in one direction, and with a decrease in temperature to below the T_g_ (removal of bias), the actuator returns back to its initial position or rest.

## 4. Conclusions

The study involves the integration of a PVDF-based unimorph actuator with a shape-memory polymer to achieve maximum bending of the cantilever actuator. From the simulation study, it was observed that the heat generated in the piezoelectric PVDF layer cannot be neglected and this heat quite significantly contributes to the total deformation of piezo-electric-based actuators. This heat is utilized to increase the maximum bending of the actuator using a prestrained SMP layer. The material properties and thickness of all the layers, the transition temperature of SMP, and the length and location of the SMP layer affect the bending of the actuator. After the experimental study, it was observed that the SMP layer mounted at the center of the actuator with a length half of the PVDF layer exhibits a maximum bending angle of 40° at a DC field of 20 V/µm after 60 s. This developed structure could be used for high unidirectional bending piezo-electric microactuators.

## Figures and Tables

**Table 1:** Properties of active and passive layers under study.

	PVDF	Scotch Tape [7]
Density (g/cc)	1.78	1.06
Elastic Modulus (GPa)	2.8	1.6
Poisson’s ratio	0.34	0.30
Electrical Conductivity (S/m)	5.56 × 10^−9^	-
Coefficient of thermal expansion (/K)	120 × 10^−6^	80 × 10^−6^

**Table 2:** Simulation results of deformation produced by unimorph due to piezoelectric and thermal effects separately.

Piezoelectric Effect		Thermal Effect	
E-field (V/µm)	Tip Deflection (mm)	Temperature at Given E-Field (K)	Tip Deflection (mm)
4	0.22	300.4	0.34
10	0.54	302.7	0.45
18	0.88	306.9	0.66
20	1.09	310.7	0.84

**Table 3:** Bending angle measured for the different samples under study.

Sample No.	Bending Angles (°)		
	Unactuated (0 V/μm)	Piezo Actuation (Immediately after 20 V/μm Is Applied to PVDF)	Enhancement by SMP Actuation ^1^ (60 s after 20 V/μm Is Applied to PVDF)
1	3	7 (Δθ = 4)	15 (Δθ = 12)
2	8	12 (Δθ = 4)	13 (Δθ = 5)
3	80	84 (Δθ = 4)	120 (Δθ = 40)

^1^ Enahncement = Total Effect − Piezo Effect − Unactuated.

**Table 4:** Comparison of different PVDF-based bending actuators.

Reference	Materials Active/Passive	Dimensions of PVDF Layer (l × w × t in mm)	Moment of Inertia (m^4^)	Max E-Field (V/µm)	Tip Deflection (mm)	Bending Curvature (1/m)
Mahale et al. [4]	PVDF	2 × 0.5 × 0.02	3.33 × 10^−19^	15	0.1	-
Liu et al. [8]	PVDF	60 × 20 × 0.16	6.83 × 10^−15^	3.75	0.3	-
Zhang et al. [7]	PVDF Terpolymer/Scotch tape	30 × 20 × 0.03	4.50 × 10^−17^	70	-	140
Ahmed et al. [6]	PVDF Terpolymer/Scotch tape	30 × 10 × 0.035	3.57 × 10^−17^	100	-	150
Present Work	PVDF/Scotch tape/SMP	20 × 2.5 × 0.05	2.60 × 10^−17^	20	0.83	104

## Data Availability

The data presented in this study are available on request from the corresponding author. The data are not publicly available due to patent pending issues.
